# Supplementary material for: Pain Treatment Strategy and Readmission Rates for Medicare Beneficiaries Post-Acute Ischemic Stroke
Source: medRxiv. 2025 Sep 27:2025.09.25.25336638. Preprint. [Version 1] doi: 10.1101/2025.09.25.25336638 (PMC12486021; doi:10.1101/2025.09.25.25336638)
Supplement: Supplement 1 — Table S1. ICD-10 diagnosis codes for Baseline Comorbidities Table S2. Medications Names Table S3. Nearest Matching Sample Characteristics Table S4. Comparison of Hazard Ratios for Initiation on Different Cohorts with Different Models Table S5. Hazard Ratios for Initiation in Stratified Analyses Figure S1 Histogram of Time to Initiation between Initiation Strategies Figure S2 Cumulative Incidence Curves with Aalen-Johansen Estimates Statistical Code [file media-1.pdf]

Supplementary Materials

Article Title:
Pain Treatment Strategy and Readmission Rates for Medicare Beneficiaries Post-Acute Ischemic Stroke

Included Materials:

Table S1. ICD-10 diagnosis codes for Baseline Comorbidities

Table S2. Medications Names

Table S3. Nearest Matching Sample Characteristics

Table S4. Comparison of Hazard Ratios for Initiation on Different Cohorts with Different Models

Table S5. Hazard Ratios for Initiation in Stratified Analyses

Figure S1 Histogram of Time to Initiation between Initiation Strategies

Figure S2 Cumulative Incidence Curves with Aalen-Johansen Estimates

Statistical Code

TABLES

Table S1. ICD-10 Codes to identify baseline characteristics.

Condition	ICD-10
Baseline Dementia	F051, G30, G311
History of MI	I252
CHF	I099, I110, I130, I132, I255, I420, I43, I50, P290, I425-I429
PVD	I70, I71, I731, I738, I739, I771, I790, I792, K551, K558, K559, Z958, Z959
CVD	G45, G46, H340, I6
COPD	I278, I279, J684, J701, J703, J40-J47, J60-J67
Paralysis	G041, G114, G801, G802, G81, G82, G839, G830-G834
Diabetes	E100, E101, E106, E108, E109, E110, E111, E116, E118, E119, E130, E131, E136, E138, E139
Renal Disease	I120, I131, N18, N19, N250, Z940, Z992, N032-N037, N052-N057, Z490-Z492
Liver Disease	I850, I859, I864, I982, K704, K711, K721, K729, K765, K766, K767
Rheumatic Disease	M05, M06, M315, M32, M33, M34, M351, M353, M360

Legend: Baseline medical conditions were filtered using the following ICD Codes. MI, Myocardial Infraction; CHF, Congestive Heart Failure; PVD, Peripheral Vascular Disease; CVD, Cardiovascular Disease; COPD, Chronic Obstructive Pulmonary Disease

Table S2. Medication Names

Brand Names	Neurontin, Gabapentin, Gralise, Horizant, Amitriptyline HCL, Perphenazine-Amitriptyline, Chlordiazepoxide-Amitriptyline, Lamotrigine ODT, Lamictal, Lamictal (Orange), Lamictal (Blue), Lamictal XR, Lamictal XR (Orange), Lamictal ODT, Lamictal ODT (Orange), Lamictal (Green), Lamotrigine ER, Lamotrigine, Lamotrigine ODT (Orange), Lamotrigine (Orange), Subvenite (Orange), Subvenite, Subvenite (Blue), Lamotrigine (Green), Lyrica, Lyrica CR, Pregabalin, Tegretol, Tegretol XR, Epitol, Carbamazepine, Carbamazepine ER, Equetro, Carbamazepine XR, Carbatrol, Dilantin, Dilantin-125, Phenytoin Sodium Extended, Phenytek, Phenytoin, Phenytoin Sodium
Generic Names	Gabapentin, Amitriptyline HCL, Perphenazine/Amitriptyline HCL, Amitriptyline/Chlordiazepoxide, Amitriptyline HCL/Chlordiazepoxide, Lamotrigine, Pregabalin, Carbamazepine, Phenytoin

Legend: Prescription drug claims were filtered using generic and brand names as displayed in the table.

Table S3. Characteristics of Patients in Nearest Matching Cohort Stratified by Initiation Strategy

Characteristics	Overall Sample	Initiation Strategy<br>Other (n=285)	Gabapentin (n=1425)	SMD
180-day Readmission* (%)	179 (10.5)	37 (13.0)	142 (10.0)	0.095
180-day Mortality* (%)	178 (10.4)	36 (12.6)	142 (10.0)	0.084
Age (median (IQR))	76 (10.75)	76 (11)	75 (11)	0.016
Age Categories (%)				
66-75	712 (41.6)	126 (44.2)	586 (41.1)	0.016
76-85	694 (40.6)	110 (38.6)	584 (41.0)	
86+	304 (17.8)	49 (17.2)	255 (17.9)	
Sex (%)				
Male	730 (42.7)	124 (43.5)	606 (42.5)	0.020
Female	980 (57.3)	161 (56.5)	819 (57.5)	
Race/Ethnicity (%)				
White	1422 (83.2)	237 (83.2)	1185 (83.2)	0.123
Black	161 (9.4)	26 (9.1)	135 (9.5)	
Asian	31 (1.8)	<11 (1.8)	>20 (1.8)	
Hispanic	38 (2.2)	<11 (2.5)	>27 (2.2)	
American Native	<11 (*)	<11 (*)	<11 (*)	
Other	20 (1.2)	<11 (1.4)	>9 (1.1)	
Unknown	29 (1.7)	<11 (2.1)	>18 (1.6)	
US Regions (%)				
Midwest	395 (25.0)	65 (24.7)	330 (25.0)	0.269
Northeast	312 (19.7)	44 (16.7)	268 (20.3)	
Southeast	430 (27.2)	92 (35.0)	338 (25.6)	
Southwest	200 (12.6)	36 (13.7)	164 (12.4)	
West	245 (15.5)	26 (9.9)	219 (16.6)	
Year of Discharge (%)				
2017	402 (23.5)	70 (24.6)	332 (23.3)	0.081
2018	345 (20.2)	50 (17.5)	295 (20.7)	
2019	317 (18.5)	55 (19.3)	262 (18.4)	
2020	343 (20.1)	58 (20.4)	285 (20.0)	
2021	303 (17.7)	52 (18.2)	251 (17.6)	
Baseline ADRD (%)	52 (3.0)	17 (6.0)	35 (2.5)	0.175
Acute MI (%)	36 (2.1)	<11 (2.5)	>25 (2.0)	0.028
History of MI (%)	42 (2.5)	<11 (2.5)	>31 (2.5)	<0.001
CHF (%)	194 (11.3)	28 (9.8)	166 (11.6)	0.059
PVD (%)	184 (10.8)	35 (12.3)	149 (10.5)	0.058
CVD (%)	181 (10.6)	42 (14.7)	139 (9.8)	0.152
COPD (%)	219 (12.8)	38 (13.3)	181 (12.7)	0.019
Hemiplegia/Paraplegia (%)	<22 (0.9)	<11 (*)	11 (0.8)	0.088
Diabetes (%)	417 (24.4)	81 (28.4)	336 (23.6)	0.111
Renal Disease (%)	213 (12.5)	34 (11.9)	179 (12.6)	0.019
Liver Disease (%)	<11 (*)	0 (0.0)	<11 (*)	0.053
Ulcers (%)	17 (1.0)	<11 (2.1)	>6 (0.8)	0.112
Rheumatic Disease (%)	67 (3.9)	11 (3.9)	56 (3.9)	0.004
Predicted mRS Categories (%)				
≤ 4	553 (32.3)	104 (36.5)	449 (31.5)	0.105
> 4	1157 (67.7)	181 (63.5)	976 (68.5)	
Length of Stay (mean (SD))	4.10 (3.76)	3.74 (3.14)	4.17 (3.87)	0.122

Legend: Characteristics of patients stratified by initiation strategy within 90 days post-discharge

*-180 days post initiation of medication
IQR, Inter-Quartile Range; ADRD, Alzheimer's Disease and Related Dementia; CHF, Congestive Heart Failure; COPD, Chronic Obstructive Pulmonary Disease; CVD, Cardiovascular Disease; MI, Myocardial Infraction; PVD, Peripheral Vascular Disease.

Table S4. Comparison of Hazard Ratios for Initiation on Different Cohorts with Different Models

**Whole Cohort**	**Readmission**	**Hazard Ratio**	**Lower 95% CI**	**Upper 95% CI**
	Unadjusted	0.676	0.412	1.109
	Adjusted (Model 1)	0.809	0.517	1.266
	Adjusted (Model 2)	0.838	0.500	1.405
	**Mortality**	**Hazard Ratio**	**Lower 95% CI**	**Upper 95% CI**
	Unadjusted	0.689	0.407	1.165
	Adjusted (Model 1)	0.726	0.449	1.176
	Adjusted (Model 2)	0.747	0.436	1.278
	**Sojourn Time**	**Hazard Ratio**	**Lower 95% CI**	**Upper 95% CI**
	Unadjusted	1.067	0.372	3.057
	Adjusted (Model 1)	1.110	0.379	3.247
	Adjusted (Model 2)	1.064	0.357	3.177
**Nearest Matching Cohort**	**Readmission**	**Hazard Ratio**	**Lower 95% CI**	**Upper 95% CI**
	Unadjusted	0.688	0.420	1.125
	Adjusted (Model 1)	0.795	0.503	1.259
	Adjusted (Model 2)	0.816	0.489	1.364
	**Mortality**	**Hazard Ratio**	**Lower 95% CI**	**Upper 95% CI**
	Unadjusted	0.682	0.404	1.152
	Adjusted (Model 1)	0.699	0.426	1.147
	Adjusted (Model 2)	0.708	0.413	1.212
	**Sojourn Time**	**Hazard Ratio**	**Lower 95% CI**	**Upper 95% CI**
	Unadjusted	1.135	0.395	3.257
	Adjusted (Model 1)	1.179	0.412	3.369
	Adjusted (Model 2)	1.100	0.369	3.281
**Exact Matching Cohort**	**Readmission**	**Hazard Ratio**	**Lower 95% CI**	**Upper 95% CI**
	Unadjusted	0.788	0.493	1.260
	Adjusted (Model 1)	0.882	0.574	1.356
	Adjusted (Model 2)	0.889	0.530	1.493
	**Mortality**	**Hazard Ratio**	**Lower 95% CI**	**Upper 95% CI**
	Unadjusted	0.665	0.401	1.102
	Adjusted (Model 1)	0.702	0.436	1.130
	Adjusted (Model 2)	0.716	0.414	1.238
	**Sojourn Time**	**Hazard Ratio**	**Lower 95% CI**	**Upper 95% CI**
	Unadjusted	1.151	0.408	3.248
	Adjusted (Model 1)	1.309	0.462	3.711
	Adjusted (Model 2)	1.230	0.414	3.653

Legend: Hazard ratios for initiating gabapentin against other medication with a follow-up period of 180 days. We compare the results of the semi-competing risks model for the whole cohort, the exact matching cohort and the nearest matching cohort. Additionally, we compare the unadjusted model with the two adjusted models described in the manuscript.

Table S5. Hazard Ratios of Initiation Strategy in Stratified Analyses

**Covariate/Stratum**	**Readmission**		**Mortality prior to Readmission**		**Mortality post Readmission**	
	**Estimate**	**95% CI**	**Estimate**	**95% CI**	**Estimate**	**95% CI**
Overall	0.871	(0.517, 1.466)	0.698	(0.402, 1.213)	1.274	(0.438, 3.708)
**Age**						
66 to 75 years	0.782	(0.360, 1.699)	0.988	(0.364, 2.681)	1.706	(0.293, 9.921)
76 to 85 years	0.606	(0.168, 2.191)	1.276	(0.365, 4.467)	0.666	(0.088, 5.061)
86 years and above	-	-	-	-	-	-
**Baseline Dementia**						
No Dementia	0.879	(0.503, 1.535)	0.703	(0.390, 1.267)	1.390	(0.432, 4.474)
Dementia	-	-	-	-	-	-
**Predicted mRS**						
≤4 (mild-to-moderate)	0.823	(0.403, 1.683)	0.674	(0.188, 2.421)	0.363	(0.087, 1.517)
>4 (high)	0.900	(0.480, 1.686)	0.640	(0.377, 1.085)	5.623	(0.533, 5.931)

Legend: Hazard ratios for initiating gabapentin against other medication with a follow-up period of 180 days in different strata of the data. Blank rows imply the semi-competing risks model did not converge.

FIGURES

Figure S1. Histogram of Time to Initiation for each Initiation Strategy

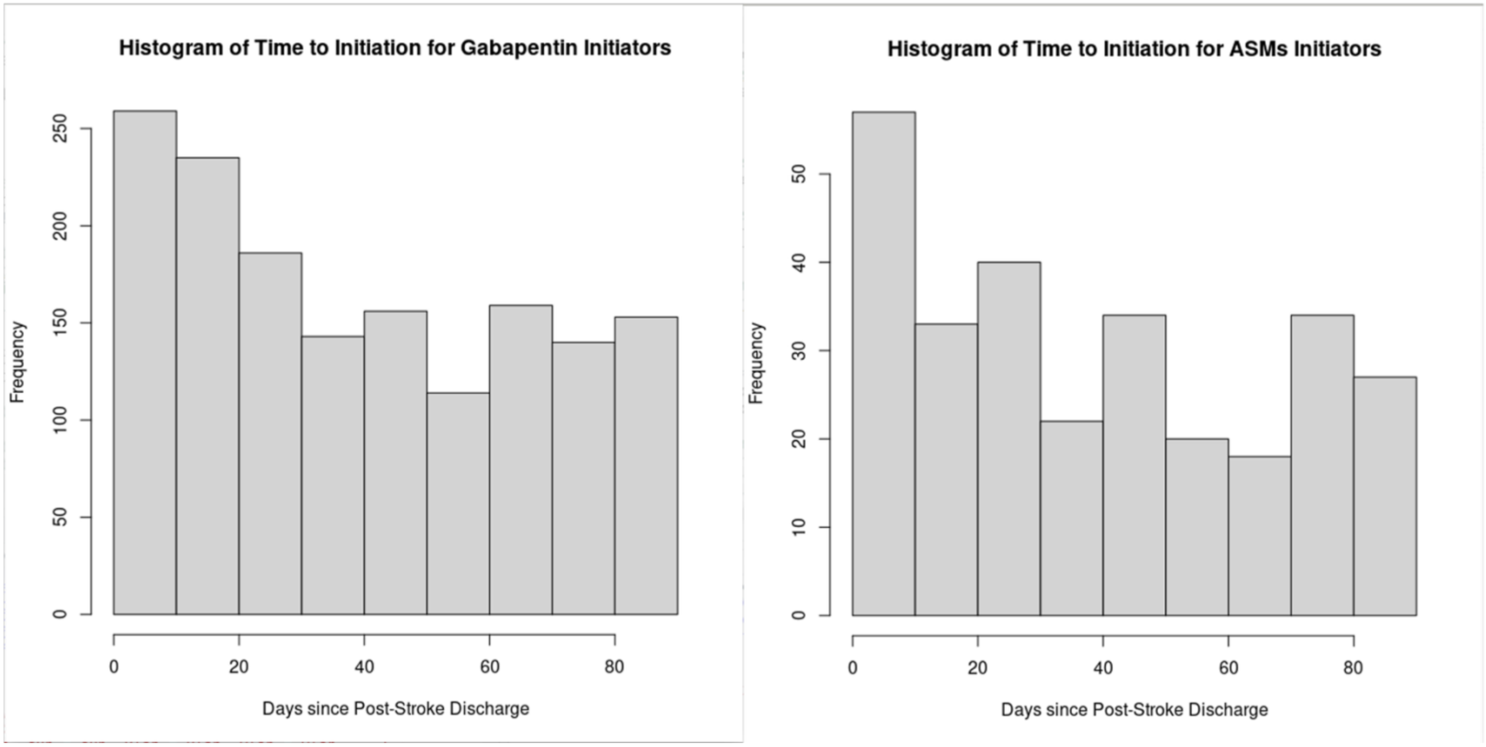

Legend: Distribution of days from discharge to medication initiation for patients who initiate gabapentin and patients initiate other medications for pain. We have labelled these as ASMs, but this also includes amitriptyline.

Figure S2. Aalen Johansen Curves for Readmission and Mortality among the Initiation Strategies

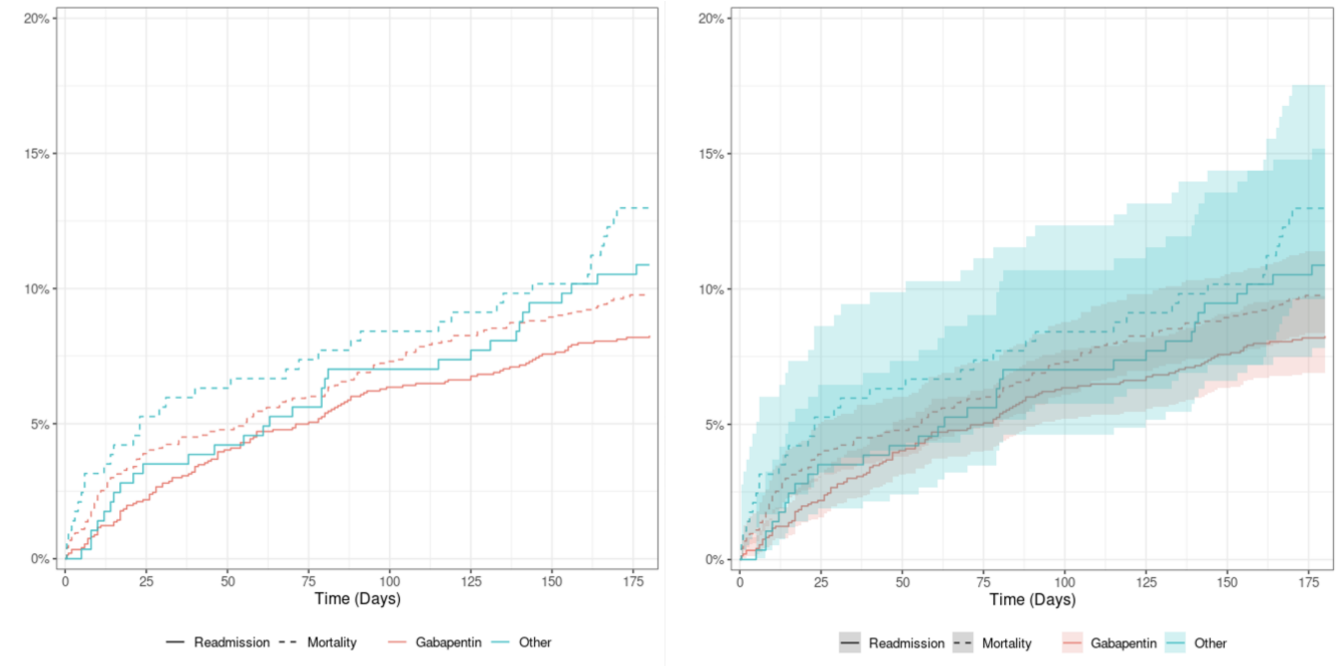

Legend: Cumulative incidence curves from the Aalen-Johansen estimates. (Left) Solid lines represent the proportion of individuals with a readmission event at the given time period from the gabapentin initiation group (given in **red**) and the other initiation group (given in **blue**). Dashed lines represent the proportion of individuals with a mortality event at the given time period from the gabapentin and the other initiation groups. (Right) Same figure as the left, with confidence intervals for the Aalen-Johansen estimates.

STATISTICAL CODE

```
#------Matching Code-----#
library("survival") #package for standard survival analysis
library("SemiCompRisks") #package for semi competing risks analysis
library(dplyr)
library(tidyverse)
library(tidyr)

# install.packages("devtools")
#devtools::install_github("harrisonreeder/SemiCompRisksFreq")
library("SemiCompRisksFreq") #package for semi competing risks analysis - from github

# example code was adapted from SemiCompRiskExamples found on github from:
# https://github.com/harrisonreeder/SemiCompRisksExamples

#### Matching ####
library(MatchIt)


#df_gab is the file with the full cohort, gabapentin is marked with 1, other with 0

df_gab_asm = df_gab_asm %>% mutate(readm_time = case_when(Readm_180_Init==1 ~ readm_day_diff-
dschg_day_diff,
                                                    Mort_180_Init==1 ~ death_day_diff-dschg_day_diff,
                                                    TRUE ~ 180),
                              mort_time = case_when(Mort_180_Init==1 ~ death_day_diff-dschg_day_diff,
                                                   TRUE ~ 180))

##Make race and comorbidities into indicator variables

df_gab_asm = df_gab_asm %>%
  mutate(race_white = as.numeric(race=="White"),
        race_asian = as.numeric(race=="Asian"),
        race_black = as.numeric(race=="Black"),
        race_hispanic = as.numeric(race=="Hispanic"),
        race_native = as.numeric(race=="North American Native"),
        race_other = as.numeric(race=="Other"),
        region_ne = case_when(is.na(USRegions)~0,
                          TRUE~as.numeric(USRegions=="Northeast")),
        dem_ind = as.numeric(dementia=="Yes"),
        cvd_ind = as.numeric(cvd=="Yes"),
        ulc_ind = as.numeric(ulcers=="Yes"))


##Standardize age

df_gab_asm = df_gab_asm %>%
  mutate(age_st = age)
```

```
df_gab_asm$age_st = (df_gab_asm$age_st-80)/10

#Nearest Matching
m.out_asm <- matchit(med_type ~ age + sex + race_white + mrs_predictions + dschg_day_diff, data = df_gab_asm,
            method = "nearest", distance = "mahalanobis", ratio = 5)
#Exact Matching
m.out_asm <- matchit(med_type ~ dschg_day_diff, data = df_gab_asm,
              method = "exact")

##Data frames
df_surv_asm <- match.data(m.out_asm)

##Correcting exceptions (same time of readmission and death)

# #Patients with readmission and mortality on the same day

same_vec = which(df_surv_asm$Readm_180_Init[df_surv_asm$readm_time==df_surv_asm$mort_time]==1)

pat_vec = which(df_surv_asm$readm_time==df_surv_asm$mort_time)[same_vec]

df_surv_asm$mort_time[pat_vec] = df_surv_asm$readm_time[pat_vec]+0.5

##Add indicators for sex


df_surv_asm = df_surv_asm %>%
  mutate(sex_ind = as.numeric(sex=="Female"))

##Adjust invalid survival times

df_surv_asm$readm_time[df_surv_asm$readm_time==0]=0.5

df_surv_asm$mort_time[df_surv_asm$mort_time==0]=0.5


##Cox PH model for mortality


#Unadjusted
model_asm = coxph(Surv(mort_time, Mort_180_Init)~med_type, data = df_surv_asm, weights = weights)

#Model 1
model_asm = coxph(Surv(mort_time, Mort_180_Init)~age + sex_ind + dem_ind + race_white + med_type +
mrs_predictions, data = df_surv_asm, weights = weights)

#Model 2
model_asm = coxph(Surv(mort_time, Mort_180_Init)~age + sex_ind + dem_ind + race_white + med_type +
mrs_predictions + dschg_day_diff + region_ne + cvd_ind, data = df_surv_asm, weights = weights)

summary(model_asm)
```

```
##Cox PH model for readmission

#Unadjusted
model_asm = coxph(Surv(readm_time, Readm_180_Init)~med_type, data = df_surv_asm, weights = weights)

#Model 1
model_asm = coxph(Surv(readm_time, Readm_180_Init)~age + sex_ind + dem_ind + race_white + med_type +
mrs_predictions, data = df_surv_asm, weights = weights)

#Model 2
model_asm = coxph(Surv(readm_time, Readm_180_Init)~age + sex_ind + dem_ind + race_white + med_type +
mrs_predictions + dschg_day_diff + region_ne + cvd_ind, data = df_surv_asm, weights = weights)

summary(model_asm)


##Testing with a semi-competing risks model
#Unadjusted
#form <- Formula::Formula(readm_time + Readm_180_Init | mort_time + Mort_180_Init ~ med_type | med_type
| med_type)
#Adjusted (Model 1)
form <- Formula::Formula(readm_time + Readm_180_Init | mort_time + Mort_180_Init ~ age + sex_ind +
dem_ind + race_white + med_type + mrs_predictions + dschg_day_diff | age + sex_ind + dem_ind + race_white +
med_type + mrs_predictions + dschg_day_diff | age + sex_ind + dem_ind + race_white + med_type +
mrs_predictions + dschg_day_diff)
#Adjusted (Model 2)
#form <- Formula::Formula(readm_time + Readm_180_Init | mort_time + Mort_180_Init ~ age + sex_ind +
dem_ind + race_white + med_type + mrs_predictions + dschg_day_diff + region_ne + cvd_ind | age + sex_ind +
dem_ind + race_white + med_type + mrs_predictions + dschg_day_diff + region_ne + cvd_ind | age + sex_ind +
dem_ind + race_white + med_type + mrs_predictions + dschg_day_diff + region_ne + cvd_ind)


fit_asm = FreqID_HReg2(Formula = form, data = df_surv_asm, model = "semi-Markov", extra_starts = 0, hazard =
"weibull", frailty = TRUE, optim_method = c("BFGS"), weights = df_surv_asm$weights)

summary(fit_asm)

#### Subsetting with age ####

##Comparison between gabapentin and other pain meds (other pain meds are cases)
df_gab_comp_age_1 = df_gab%>%
  filter(med_type>0, age<=75)%>%
  mutate(med_type=1-as.integer(med_type/2))

df_gab_comp_age_2 = df_gab%>%
  filter(med_type>0, age>75, age<=85)%>%
  mutate(med_type=1-as.integer(med_type/2))

df_gab_comp_age_3 = df_gab%>%
```

```
  filter(med_type>0, age>85)%>%
  mutate(med_type=1-as.integer(med_type/2))

df_gab_comp_age_1 = df_gab_comp_age_1 %>% mutate(readm_time = case_when(Readm_180_Init==1 ~
readm_day_diff-dschg_day_diff,
                                                                        Mort_180_Init==1 ~ death_day_diff-dschg_day_diff,
                                                                        TRUE ~ 180),
                                                 mort_time = case_when(Mort_180_Init==1 ~ death_day_diff-dschg_day_diff,
                                                                       TRUE ~ 180))
df_gab_comp_age_2 = df_gab_comp_age_2 %>% mutate(readm_time = case_when(Readm_180_Init==1 ~
readm_day_diff-dschg_day_diff,
                                                                        Mort_180_Init==1 ~ death_day_diff-dschg_day_diff,
                                                                        TRUE ~ 180),
                                                 mort_time = case_when(Mort_180_Init==1 ~ death_day_diff-dschg_day_diff,
                                                                       TRUE ~ 180))
df_gab_comp_age_3 = df_gab_comp_age_3 %>% mutate(readm_time = case_when(Readm_180_Init==1 ~
readm_day_diff-dschg_day_diff,
                                                                        Mort_180_Init==1 ~ death_day_diff-dschg_day_diff,
                                                                        TRUE ~ 180),
                                                 mort_time = case_when(Mort_180_Init==1 ~ death_day_diff-dschg_day_diff,
                                                                       TRUE ~ 180))

##Make race and comorbidities into indicator variables

df_gab_comp_age_1 = df_gab_comp_age_1 %>%
  mutate(race_white = as.numeric(race=="White"),
         race_asian = as.numeric(race=="Asian"),
         race_black = as.numeric(race=="Black"),
         race_hispanic = as.numeric(race=="Hispanic"),
         race_native = as.numeric(race=="North American Native"),
         race_other = as.numeric(race=="Other"),
         region_ne = case_when(is.na(USRegions)~0,
                               TRUE~as.numeric(USRegions=="Northeast")),
         dem_ind = as.numeric(dementia=="Yes"),
         cvd_ind = as.numeric(cvd=="Yes"),
         ulc_ind = as.numeric(ulcers=="Yes"))

df_gab_comp_age_2 = df_gab_comp_age_2 %>%
  mutate(race_white = as.numeric(race=="White"),
         race_asian = as.numeric(race=="Asian"),
         race_black = as.numeric(race=="Black"),
         race_hispanic = as.numeric(race=="Hispanic"),
         race_native = as.numeric(race=="North American Native"),
         race_other = as.numeric(race=="Other"),
         region_ne = case_when(is.na(USRegions)~0,
                               TRUE~as.numeric(USRegions=="Northeast")),
         dem_ind = as.numeric(dementia=="Yes"),
         cvd_ind = as.numeric(cvd=="Yes"),
         ulc_ind = as.numeric(ulcers=="Yes"))
```

```
df_gab_comp_age_3 = df_gab_comp_age_3 %>%
  mutate(race_white = as.numeric(race=="White"),
        race_asian = as.numeric(race=="Asian"),
        race_black = as.numeric(race=="Black"),
        race_hispanic = as.numeric(race=="Hispanic"),
        race_native = as.numeric(race=="North American Native"),
        race_other = as.numeric(race=="Other"),
        region_ne = case_when(is.na(USRegions)~0,
                        TRUE~as.numeric(USRegions=="Northeast")),
        dem_ind = as.numeric(dementia=="Yes"),
        cvd_ind = as.numeric(cvd=="Yes"),
        ulc_ind = as.numeric(ulcers=="Yes"))

#Nearest Matching
m.out_comp_age_1 <- matchit(med_type ~ sex + race_white + dschg_day_diff + mrs_predictions, data =
df_gab_comp_age_1,
                 method = "nearest", distance = "mahalanobis", ratio = 5)
m.out_comp_age_2 <- matchit(med_type ~ sex + race_white + dschg_day_diff + mrs_predictions, data =
df_gab_comp_age_2,
                  method = "nearest", distance = "mahalanobis", ratio = 5)
m.out_comp_age_3 <- matchit(med_type ~ sex + race_white + dschg_day_diff + mrs_predictions, data =
df_gab_comp_age_3,
                       method = "nearest", distance = "mahalanobis", ratio = 4)
#Exact Matching
m.out_comp_age_1 <- matchit(med_type ~ dschg_day_diff, data = df_gab_comp_age_1,
                 method = "exact")
m.out_comp_age_2 <- matchit(med_type ~ dschg_day_diff, data = df_gab_comp_age_2,
                  method = "exact")
m.out_comp_age_3 <- matchit(med_type ~ dschg_day_diff, data = df_gab_comp_age_3,
                       method = "exact")

##Data frames
df_surv_comp_age_1 <- match.data(m.out_comp_age_1)
df_surv_comp_age_2 <- match.data(m.out_comp_age_2)
df_surv_comp_age_3 <- match.data(m.out_comp_age_3)

##Correcting exceptions (same time of readmission and death)

#Patients with readmission and mortality on the same day
same_vec =
which(df_surv_comp_age_1$Readm_180_Init[df_surv_comp_age_1$readm_time==df_surv_comp_age_1$mort_t
ime]==1)

pat_vec = which(df_surv_comp_age_1$readm_time==df_surv_comp_age_1$mort_time)[same_vec]

#Add offset for mortality
df_surv_comp_age_1$mort_time[pat_vec] = df_surv_comp_age_1$readm_time[pat_vec]+0.5
```

```
same_vec =
which(df_surv_comp_age_2$Readm_180_Init[df_surv_comp_age_2$readm_time==df_surv_comp_age_2$mort_t
ime]==1)

pat_vec = which(df_surv_comp_age_2$readm_time==df_surv_comp_age_2$mort_time)[same_vec]

df_surv_comp_age_2$mort_time[pat_vec] = df_surv_comp_age_2$readm_time[pat_vec]+0.5

same_vec =
which(df_surv_comp_age_3$Readm_180_Init[df_surv_comp_age_3$readm_time==df_surv_comp_age_3$mort_t
ime]==1)

pat_vec = which(df_surv_comp_age_3$readm_time==df_surv_comp_age_3$mort_time)[same_vec]

df_surv_comp_age_3$mort_time[pat_vec] = df_surv_comp_age_3$readm_time[pat_vec]+0.5

##Add indicators for sex and recode med type

df_surv_comp_age_1 = df_surv_comp_age_1 %>%
  mutate(sex_ind = as.numeric(sex=="Female"),
        med_type = 1 - med_type)

df_surv_comp_age_2 = df_surv_comp_age_2 %>%
  mutate(sex_ind = as.numeric(sex=="Female"),
        med_type = 1 - med_type)

df_surv_comp_age_3 = df_surv_comp_age_3 %>%
  mutate(sex_ind = as.numeric(sex=="Female"),
        med_type = 1 - med_type)

##Adjust invalid survival times

df_surv_comp_age_1$readm_time[df_surv_comp_age_1$readm_time==0]=0.5

df_surv_comp_age_2$readm_time[df_surv_comp_age_2$readm_time==0]=0.5

df_surv_comp_age_3$readm_time[df_surv_comp_age_3$readm_time==0]=0.5

df_surv_comp_age_1$mort_time[df_surv_comp_age_1$mort_time==0]=0.5

df_surv_comp_age_2$mort_time[df_surv_comp_age_2$mort_time==0]=0.5

df_surv_comp_age_3$mort_time[df_surv_comp_age_3$mort_time==0]=0.5


##Mortality analysis

model_asm_age_1 = coxph(Surv(mort_time, Mort_180_Init)~med_type + dschg_day_diff + mrs_predictions, data
= df_surv_comp_age_1)
```

```
summary(model_asm_age_1)

model_asm_age_2 = coxph(Surv(mort_time, Mort_180_Init)~med_type + dschg_day_diff + mrs_predictions, data
= df_surv_comp_age_2)

summary(model_asm_age_2)

model_asm_age_3 = coxph(Surv(mort_time, Mort_180_Init)~med_type + dschg_day_diff + mrs_predictions, data
= df_surv_comp_age_3)

summary(model_asm_age_3)

##Readmission analysis

model_asm_age_1 = coxph(Surv(readm_time, Readm_180_Init)~sex_ind + dem_ind + race_white + med_type +
mrs_predictions, data = df_surv_comp_age_1, weights = weights)

summary(model_asm_age_1)

model_asm_age_2 = coxph(Surv(readm_time, Readm_180_Init)~sex_ind + dem_ind + race_white + med_type +
mrs_predictions, data = df_surv_comp_age_2, weights = weights)

summary(model_asm_age_2)

model_asm_age_3 = coxph(Surv(readm_time, Readm_180_Init)~sex_ind + dem_ind + race_white + med_type +
mrs_predictions, data = df_surv_comp_age_3, weights = weights)

summary(model_asm_age_3)

##Testing with a semi-competing risks model
#Unadjusted
form <- Formula::Formula(readm_time + Readm_180_Init | mort_time + Mort_180_Init ~ med_type | med_type |
med_type)
#Adjusted
form <- Formula::Formula(readm_time + Readm_180_Init | mort_time + Mort_180_Init ~ sex_ind + dem_ind +
race_white + med_type + mrs_predictions + dschg_day_diff | sex_ind + race_white + med_type + mrs_predictions
+ dschg_day_diff | sex_ind + race_white + med_type + mrs_predictions + dschg_day_diff)

fit_comp_age_1 = FreqID_HReg2(Formula = form, data = df_surv_comp_age_1, model = "semi-Markov",
extra_starts = 0, hazard = "weibull", frailty = TRUE, optim_method = c("BFGS"))

fit_comp_age_2 = FreqID_HReg2(Formula = form, data = df_surv_comp_age_2, model = "semi-Markov",
extra_starts = 0, hazard = "weibull", frailty = TRUE, optim_method = c("BFGS"))

fit_comp_age_3 = FreqID_HReg2(Formula = form, data = df_surv_comp_age_3, model = "semi-Markov",
extra_starts = 0, hazard = "weibull", frailty = TRUE, optim_method = c("BFGS"))

summary(fit_comp_age_3)
```

```
#### Subsetting with dementia ####

##Comparison between gabapentin and other pain meds (other pain meds are cases)
df_gab_comp_dem = df_gab%>%
  filter(med_type>0, dementia=="Yes")%>%
  mutate(med_type=1-as.integer(med_type/2))

df_gab_comp_no_dem = df_gab%>%
  filter(med_type>0, dementia=="No")%>%
  mutate(med_type=1-as.integer(med_type/2))

df_gab_comp_dem = df_gab_comp_dem %>% mutate(readm_time = case_when(Readm_180_Init==1 ~
readm_day_diff-dschg_day_diff,
                                                Mort_180_Init==1 ~ death_day_diff-dschg_day_diff,
                                                TRUE ~ 180),
                                 mort_time = case_when(Mort_180_Init==1 ~ death_day_diff-dschg_day_diff,
                                                TRUE ~ 180))
df_gab_comp_no_dem = df_gab_comp_no_dem %>% mutate(readm_time = case_when(Readm_180_Init==1 ~
readm_day_diff-dschg_day_diff,
                                                Mort_180_Init==1 ~ death_day_diff-dschg_day_diff,
                                                TRUE ~ 180),
                                 mort_time = case_when(Mort_180_Init==1 ~ death_day_diff-dschg_day_diff,
                                                TRUE ~ 180))

##Make race and comorbidities into indicator variables

df_gab_comp_dem = df_gab_comp_dem %>%
  mutate(race_white = as.numeric(race=="White"),
         race_asian = as.numeric(race=="Asian"),
         race_black = as.numeric(race=="Black"),
         race_hispanic = as.numeric(race=="Hispanic"),
         race_native = as.numeric(race=="North American Native"),
         race_other = as.numeric(race=="Other"),
         region_ne = case_when(is.na(USRegions)~0,
                     TRUE~as.numeric(USRegions=="Northeast")),
         dem_ind = as.numeric(dementia=="Yes"),
         cvd_ind = as.numeric(cvd=="Yes"),
         ulc_ind = as.numeric(ulcers=="Yes"))

df_gab_comp_no_dem = df_gab_comp_no_dem %>%
  mutate(race_white = as.numeric(race=="White"),
         race_asian = as.numeric(race=="Asian"),
         race_black = as.numeric(race=="Black"),
         race_hispanic = as.numeric(race=="Hispanic"),
         race_native = as.numeric(race=="North American Native"),
         race_other = as.numeric(race=="Other"),
         region_ne = case_when(is.na(USRegions)~0,
                     TRUE~as.numeric(USRegions=="Northeast")),
         dem_ind = as.numeric(dementia=="Yes"),
```

```
        cvd_ind = as.numeric(cvd=="Yes"),
        ulc_ind = as.numeric(ulcers=="Yes"))

#Nearest Matching
m.out_comp_dem <- matchit(med_type ~ sex + race_white + dschg_day_diff + mrs_predictions, data =
df_gab_comp_dem,
                        method = "nearest", distance = "mahalanobis", ratio = 2)
m.out_comp_no_dem <- matchit(med_type ~ sex + race_white + dschg_day_diff + mrs_predictions, data =
df_gab_comp_no_dem,
                        method = "nearest", distance = "mahalanobis", ratio = 5)
#Exact Matching
m.out_comp_dem <- matchit(med_type ~ dschg_day_diff, data = df_gab_comp_dem,
                        method = "exact")
m.out_comp_no_dem <- matchit(med_type ~ dschg_day_diff, data = df_gab_comp_no_dem,
                        method = "exact")

##Data frames
df_surv_comp_dem <- match.data(m.out_comp_dem)
df_surv_comp_no_dem <- match.data(m.out_comp_no_dem)

##Correcting exceptions (same time of readmission and death)

#Patients with readmission and mortality on the same day
same_vec =
which(df_surv_comp_dem$Readm_180_Init[df_surv_comp_dem$readm_time==df_surv_comp_dem$mort_time]
==1)

pat_vec = which(df_surv_comp_dem$readm_time==df_surv_comp_dem$mort_time)[same_vec]

#Add offset for mortality
df_surv_comp_dem$mort_time[pat_vec] = df_surv_comp_dem$readm_time[pat_vec]+0.5

same_vec =
which(df_surv_comp_no_dem$Readm_180_Init[df_surv_comp_no_dem$readm_time==df_surv_comp_no_dem$
mort_time]==1)

pat_vec = which(df_surv_comp_no_dem$readm_time==df_surv_comp_no_dem$mort_time)[same_vec]

df_surv_comp_no_dem$mort_time[pat_vec] = df_surv_comp_no_dem$readm_time[pat_vec]+0.5

##Add indicators for sex and recode med type

df_surv_comp_dem = df_surv_comp_dem %>%
  mutate(sex_ind = as.numeric(sex=="Female"),
        med_type = 1 - med_type)

df_surv_comp_no_dem = df_surv_comp_no_dem %>%
  mutate(sex_ind = as.numeric(sex=="Female"),
        med_type = 1 - med_type)
```

```
##Adjust invalid survival times

df_surv_comp_dem$readm_time[df_surv_comp_dem$readm_time==0]=0.5

df_surv_comp_no_dem$readm_time[df_surv_comp_no_dem$readm_time==0]=0.5

df_surv_comp_dem$mort_time[df_surv_comp_dem$mort_time==0]=0.5

df_surv_comp_no_dem$mort_time[df_surv_comp_no_dem$mort_time==0]=0.5

##Mortality analysis

model_asm_no_dem = coxph(Surv(mort_time, Mort_180_Init)~med_type + dschg_day_diff + mrs_predictions,
data = df_surv_comp_no_dem)

summary(model_asm_no_dem)

model_asm_dem = coxph(Surv(mort_time, Mort_180_Init)~med_type + dschg_day_diff + mrs_predictions, data =
df_surv_comp_dem)

summary(model_asm_dem)

##Readmission analysis

model_asm_no_dem = coxph(Surv(readm_time, Readm_180_Init)~age + sex_ind + race_white + med_type +
mrs_predictions, data = df_surv_comp_no_dem, weights = weights)

summary(model_asm_no_dem)

model_asm_dem = coxph(Surv(readm_time, Readm_180_Init)~age + sex_ind + race_white + med_type +
mrs_predictions, data = df_surv_comp_dem, weights = weights)

summary(model_asm_dem)

##Testing with a semi-competing risks model
#Unadjusted
form <- Formula::Formula(readm_time + Readm_180_Init | mort_time + Mort_180_Init ~ med_type | med_type |
med_type)
#Adjusted
form <- Formula::Formula(readm_time + Readm_180_Init | mort_time + Mort_180_Init ~ age + sex_ind +
race_white + med_type + mrs_predictions + dschg_day_diff | age + sex_ind + race_white + med_type +
mrs_predictions + dschg_day_diff | age + sex_ind + race_white + med_type + mrs_predictions + dschg_day_diff)

fit_comp_dem = FreqID_HReg2(Formula = form, data = df_surv_comp_dem, model = "semi-Markov", extra_starts
= 0, hazard = "weibull", frailty = TRUE, optim_method = c("BFGS"))

fit_comp_no_dem = FreqID_HReg2(Formula = form, data = df_surv_comp_no_dem, model = "semi-Markov",
extra_starts = 0, hazard = "weibull", frailty = TRUE, optim_method = c("BFGS"))

summary(fit_comp_no_dem)
```

```
#### Subsetting with MRSScore ####

##Comparison between gabapentin and other pain meds (other pain meds are cases)
df_gab_comp_low_mrs = df_gab%>%
  filter(med_type>0, mrs_predictions==0)%>%
  mutate(med_type=1-as.integer(med_type/2))

df_gab_comp_high_mrs = df_gab%>%
  filter(med_type>0, mrs_predictions==1)%>%
  mutate(med_type=1-as.integer(med_type/2))

df_gab_comp_low_mrs = df_gab_comp_low_mrs %>% mutate(readm_time = case_when(Readm_180_Init==1 ~
readm_day_diff-dschg_day_diff,
                                                Mort_180_Init==1 ~ death_day_diff-dschg_day_diff,
                                                TRUE ~ 180),
                                     mort_time = case_when(Mort_180_Init==1 ~ death_day_diff-dschg_day_diff,
                                                TRUE ~ 180))
df_gab_comp_high_mrs = df_gab_comp_high_mrs %>% mutate(readm_time = case_when(Readm_180_Init==1 ~
readm_day_diff-dschg_day_diff,
                                                 Mort_180_Init==1 ~ death_day_diff-dschg_day_diff,
                                                 TRUE ~ 180),
                                      mort_time = case_when(Mort_180_Init==1 ~ death_day_diff-dschg_day_diff,
                                                 TRUE ~ 180))

##Make race and comorbidities into indicator variables

df_gab_comp_low_mrs = df_gab_comp_low_mrs %>%
  mutate(race_white = as.numeric(race=="White"),
        race_asian = as.numeric(race=="Asian"),
        race_black = as.numeric(race=="Black"),
        race_hispanic = as.numeric(race=="Hispanic"),
        race_native = as.numeric(race=="North American Native"),
        race_other = as.numeric(race=="Other"),
        region_ne = case_when(is.na(USRegions)~0,
                    TRUE~as.numeric(USRegions=="Northeast")),
        dem_ind = as.numeric(dementia=="Yes"),
        cvd_ind = as.numeric(cvd=="Yes"),
        ulc_ind = as.numeric(ulcers=="Yes"))

df_gab_comp_high_mrs = df_gab_comp_high_mrs %>%
  mutate(race_white = as.numeric(race=="White"),
        race_asian = as.numeric(race=="Asian"),
        race_black = as.numeric(race=="Black"),
        race_hispanic = as.numeric(race=="Hispanic"),
        race_native = as.numeric(race=="North American Native"),
        race_other = as.numeric(race=="Other"),
        region_ne = case_when(is.na(USRegions)~0,
                    TRUE~as.numeric(USRegions=="Northeast")),
```

```
        dem_ind = as.numeric(dementia=="Yes"),
        cvd_ind = as.numeric(cvd=="Yes"),
        ulc_ind = as.numeric(ulcers=="Yes"))


#Nearest Matching
m.out_comp_low_mrs <- matchit(med_type ~ sex + race_white + dschg_day_diff + mrs_predictions, data =
df_gab_comp_low_mrs,
                      method = "nearest", distance = "mahalanobis", ratio = 4)
m.out_comp_high_mrs <- matchit(med_type ~ sex + race_white + dschg_day_diff + mrs_predictions, data =
df_gab_comp_high_mrs,
                        method = "nearest", distance = "mahalanobis", ratio = 5)
#Exact Matching
m.out_comp_low_mrs <- matchit(med_type ~ dschg_day_diff, data = df_gab_comp_low_mrs,
                      method = "exact")
m.out_comp_high_mrs <- matchit(med_type ~ dschg_day_diff, data = df_gab_comp_high_mrs,
                        method = "exact")

##Data frames
df_surv_comp_low_mrs <- match.data(m.out_comp_low_mrs)
df_surv_comp_high_mrs <- match.data(m.out_comp_high_mrs)

##Correcting exceptions (same time of readmission and death)

#Patients with readmission and mortality on the same day
same_vec =
which(df_surv_comp_low_mrs$Readm_180_Init[df_surv_comp_low_mrs$readm_time==df_surv_comp_low_mrs
$mort_time]==1)

pat_vec = which(df_surv_comp_low_mrs$readm_time==df_surv_comp_low_mrs$mort_time)[same_vec]

#Add offset for mortality
df_surv_comp_low_mrs$mort_time[pat_vec] = df_surv_comp_low_mrs$readm_time[pat_vec]+0.5

same_vec =
which(df_surv_comp_high_mrs$Readm_180_Init[df_surv_comp_high_mrs$readm_time==df_surv_comp_high_m
rs$mort_time]==1)

pat_vec = which(df_surv_comp_high_mrs$readm_time==df_surv_comp_high_mrs$mort_time)[same_vec]

df_surv_comp_high_mrs$mort_time[pat_vec] = df_surv_comp_high_mrs$readm_time[pat_vec]+0.5

##Add indicators for sex and recode med type

df_surv_comp_low_mrs = df_surv_comp_low_mrs %>%
  mutate(sex_ind = as.numeric(sex=="Female"),
        med_type = 1 - med_type)

df_surv_comp_high_mrs = df_surv_comp_high_mrs %>%
  mutate(sex_ind = as.numeric(sex=="Female"),
```

```
        med_type = 1 - med_type)

##Adjust invalid survival times

df_surv_comp_low_mrs$readm_time[df_surv_comp_low_mrs$readm_time==0]=0.5

df_surv_comp_high_mrs$readm_time[df_surv_comp_high_mrs$readm_time==0]=0.5

df_surv_comp_low_mrs$mort_time[df_surv_comp_low_mrs$mort_time==0]=0.5

df_surv_comp_high_mrs$mort_time[df_surv_comp_high_mrs$mort_time==0]=0.5

##Mortality analysis

model_asm_high_mrs = coxph(Surv(mort_time, Mort_180_Init)~med_type + dschg_day_diff + mrs_predictions,
data = df_surv_comp_high_mrs)

summary(model_asm_high_mrs)

model_asm_low_mrs = coxph(Surv(mort_time, Mort_180_Init)~med_type + dschg_day_diff + mrs_predictions,
data = df_surv_comp_low_mrs)

summary(model_asm_low_mrs)

##Readmission analysis

model_asm_high_mrs = coxph(Surv(readm_time, Readm_180_Init)~age + sex_ind + dem_ind + race_white +
med_type, data = df_surv_comp_high_mrs, weights = weights)

summary(model_asm_high_mrs)

model_asm_low_mrs = coxph(Surv(readm_time, Readm_180_Init)~age + sex_ind + dem_ind + race_white +
med_type, data = df_surv_comp_low_mrs, weights = weights)

summary(model_asm_low_mrs)

##Testing with a semi-competing risks model
#Unadjusted
form <- Formula::Formula(readm_time + Readm_180_Init | mort_time + Mort_180_Init ~ med_type | med_type |
med_type)
#Adjusted
form <- Formula::Formula(readm_time + Readm_180_Init | mort_time + Mort_180_Init ~ med_type +
dschg_day_diff | med_type + dschg_day_diff | med_type + dschg_day_diff)

fit_comp_low_mrs = FreqID_HReg2(Formula = form, data = df_surv_comp_low_mrs, model = "semi-Markov",
extra_starts = 0, hazard = "weibull", frailty = TRUE, optim_method = c("BFGS"))

fit_comp_high_mrs = FreqID_HReg2(Formula = form, data = df_surv_comp_high_mrs, model = "semi-Markov",
extra_starts = 0, hazard = "weibull", frailty = TRUE, optim_method = c("BFGS"))
```

```
summary(fit_comp_high_mrs)
```
